# Stertorous Breathing in Apoplexy

**Published:** 1881-12

**Authors:** 


### STERTOROUS BREATHING IN APOPLEXY AND THE MANAGEMENT OF THF APOPLECTIC STATE.

In a paper on this subject in the *Brit. Med. Jour.*, for May 28, 1881, Dr. Robert Bowles says that the causes of stertorous breathing in apoplexy are mechanical, and can at all times be so changed as to alter altogether the nature of a case, and often make the difference of recovery or death. The truth is, two separate conditions of the apoplectic state have been jumbled together and treated as one—the cerebral affection and the condition of suffocation consequent upon it.

Stertor in one sense is but a croup in the pharynx, or apoplexy plus suffocation, as croup is laryngitis plus suffocation.

We feel it necessary to relieve croup by a serious operation; whereas stertor is left to itself, although it may be relieved by merely changing the position of the body.

Dr. Bowles divides the various forms of stertor commonly observed as follows:

1. Nasal Stertor arises from paralysis of the nerves supplying the elevators and dilators of the alæ nasi, so that the in-going air, as in sniffling, draws the alæ nasi toward the septum, and sometimes causes a serious obstruction to the breathing, and certainly hastens death, as well as needlessly distresses the bystanding and sorrowing relatives. It is often a symptom of the gravest kind; is unaffected by the position of the body, but may always be relieved by mechanical means.

2. Palatine Stertor occurs when the air in rushing through the nose or mouth causes a vibration of the soft palate. It is usually of the least consequence; i. e., it obstructs the breathing only very partially, and cannot always be removed by changing the position of the body. It is affected by the size of the tongue, the length of the uvula, the position of the chin, and other incidental conditions, all of which may be obviated if the obstruction to the breathing be sufficient to render it worth the doing.

3. Pharyngeal Stertor is the most common in severe cases of apoplexy, when patients are recumbent. This may always be obviated by properly arranging the position of the patient, allowing the paralyzed mass—the tongue—to gravitate to one side rather than against the back of the pharynx.

4. Mucous Stertor, when unconnected with lung-engorgement, the consequence of suffocation from stertor, occurs only in very serious cases, depending upon interference with the nutritive processes of the lung tissues, probably arising remotely from accident to or pressure upon the medulla oblongata. This can always be satisfactorily removed by proper attention to the position of the body.

These principles apply not merely to apoplexy, but also to all apoplectic conditions. Especially may be mentioned drowning, epilepsy, convulsions in children, meningitis with effusion, death-rattles, fracture of the skull, concussion, bronchitis, especially that of old people, sudden œdema of the lungs, large hemorrhage from the lungs, and also all conditions allied to the apoplectic, whether there be mucus or not.

